# Atlantic Deep-water Response to the Early Pliocene Shoaling of the Central American Seaway

**DOI:** 10.1038/srep12252

**Published:** 2015-07-20

**Authors:** David B. Bell, Simon J. A. Jung, Dick Kroon, David A. Hodell, Lucas J. Lourens, Maureen E. Raymo

**Affiliations:** 1 School of Geosciences, University of Edinburgh, Edinburgh, UK; 2 Department of Earth Science, University of Cambridge, Cambridge, UK; 3 Faculty of Geosciences, Utrecht University, Utrecht, Netherlands; 4 Lamont Doherty Earth Observatory, Columbia University, New York, USA

## Abstract

The early Pliocene shoaling of the Central American Seaway (CAS), ~4.7–4.2 million years ago (mega annum-Ma), is thought to have strengthened Atlantic Meridional Overturning Circulation (AMOC). The associated increase in northward flux of heat and moisture may have significantly influenced the evolution of Pliocene climate. While some evidence for the predicted increase in North Atlantic Deep Water (NADW) formation exists in the Caribbean and Western Atlantic, similar evidence is missing in the wider Atlantic. Here, we present stable carbon (δ^13^C) and oxygen (δ^18^O) isotope records from the Southeast Atlantic-a key region for monitoring the southern extent of NADW. Using these data, together with other δ^13^C and δ^18^O records from the Atlantic, we assess the impact of the early Pliocene CAS shoaling phase on deep-water circulation. We find that NADW formation was vigorous prior to 4.7 Ma and showed limited subsequent change. Hence, the overall structure of the deep Atlantic was largely unaffected by the early Pliocene CAS shoaling, corroborating other evidence that indicates larger changes in NADW resulted from earlier and deeper shoaling phases. This finding implies that the early Pliocene shoaling of the CAS had no profound impact on the evolution of climate.

Changes in major ocean-gateways during the late Neogene are considered to have had far-reaching impacts on the evolution of climate, mediated through large-scale changes in ocean circulation. Notably, it has been proposed that the progressive closure of the CAS, which occurred during the Pliocene^1^, was a necessary precondition for establishing enhanced (modern-like) Atlantic Meridional Overturning Circulation (AMOC) conditions^2^,^3^. The so-called “Panama Closure Hypothesis” posits that as the CAS closed, diminishing upper-water exchange between the Pacific Ocean and Caribbean Sea led to enhanced flow of warm and salty surface water into the North Atlantic via the Gulf Stream^2^,^3^. The additional salt carried northward would have increased density in the surface waters of deep-water formation regions, such as the Nordic and Labrador Seas. In turn, this is expected to have led to stronger NADW formation, driving enhanced AMOC along with a marked reorganization of the deep-water flow in the Atlantic Ocean^3^,^4^. Such closure induced changes have been implicated in enhancing ice sheet growth and promoting the onset of Northern Hemisphere Glaciation through an increase in the North Atlantic moisture budget^5^,^6^,^7^, as well as in triggering an overall shoaling of the tropical thermocline and promoting the development of the Eastern Equatorial Pacific cold tongue^8^. In contrast, it has also been argued that the resulting increase in northward heat flux hindered ice sheet growth and delayed Northern Hemisphere Glaciation^9^,^10^.

Elucidating the timing and nature of the CAS closure is crucial for evaluating its impact on AMOC and climate. However, geological records reveal it as a complex process, with many details remaining unclear and contested^11^,^12^,^13^,^14^,^15^,^16^. The timeline of closure events is estimated to range from the emergence of land in the CAS as early as ~15–20 Ma^17^,^18^, to closure of the deep-water connection by ~7–10 Ma^19^,^20^, and complete restriction of Pacific-Caribbean surface-water exchange between ~3.5–2.5 Ma^12^. In addition, while there is strong general agreement between modeling studies that AMOC strengthens in response to CAS closure, there is a wide range in the magnitude of AMOC response, especially for the final few hundred meters of shoaling (for an overview, see^20^ and references therein). Hence, both the anticipated timing and the magnitude of CAS induced changes in AMOC are uncertain.

Nevertheless, arguably the clearest and best-constrained evidence for the final stages of CAS closure, particularly with regards to its impact on regional surface-water oceanography, is in the development of a permanent inter-basin surface-water δ^18^O-gradient between the Pacific and Caribbean Sea (Fig. 1). An initial δ^18^O-gradient occurred as early as ~4.7 Ma, followed by a strengthening between 4.4–4.2 Ma, implying restricted upper-water exchange across the CAS^2^,^21^,^22^. In line with the δ^18^O data, Mg/Ca analyses on planktonic foraminifera indicate a build-up of heat and salinity in the Caribbean^8^,^22^,^23^. Haug and Tiedemann^3^ suggested that this interval reflects a major phase in the final closure of the seaway, with shoaling to within 100 m water depth by 4.2 Ma. More recently, however, Mestas-Nuñez and Molnar^13^ have suggested that these changes in the Caribbean may be unrelated to CAS shoaling: Instead, they propose it may be a consequence of a shifting dominance of El Niño- to La Niña-like conditions in the eastern tropical Pacific. Regardless of the cause, the build-up of heat and salinity in Caribbean surface waters during the period ~4.7–4.2 Ma is uncontested and, as a consequence, the expectation of an increase in AMOC remains. Hence, in this study, the early Pliocene shoaling of the CAS refers to these well-documented changes in Caribbean surface-water oceanography.

Based on the original interpretation of surface water changes in the Caribbean, many studies have marked the early Pliocene as a key interval in the CAS closure history and linked it to proxy records that indicate an enhancement in NADW^3^,^4^,^5^,^8^,^19^,^21^,^24^,^25^. However, the evidence for NADW changes during the early Pliocene is mainly limited to the Caribbean Sea and deep equatorial Western Atlantic (Fig. 2). As both bathymetric constraints from major ocean ridges and differences between water mass advection within the Deep Western Boundary Current (DWBC) and interior pathways are markedly influential in setting up gradients and pathways within the Atlantic^26^,^27^,^28^ (Fig. 3), it is straightforward to envisage scenarios whereby regional changes in water mass prevalence are not representative of large-scale changes in the wider deep Atlantic. Indeed, important regional differences in Pleistocene deep-water circulation have been shown previously^29^,^30^,^31^,^32^,^33^, reinforcing the need to consider previously documented changes in a wider spatial context.

In light of these considerations, we find that evidence for large-scale changes in AMOC during the early Pliocene is hitherto unclear. In particular, changes in the southern extent of NADW—an important indicator of AMOC—are so far not well sampled. In the Atlantic sector of the Southern Ocean, low resolution and poorly time-constrained benthic stable isotope records exist for Ocean Drilling Program (ODP) Sites 1088^34^,^35^ (2080 m depth, 41°S, 14°E) and 704^36^ (2530 m depth, 47°S, 7°E), but these lie within a region of complex ocean mixing, with time-varying contributions from Circumpolar Deep Water and Antarctic Intermediate Water. Indeed, questions have arisen over the origin of the signal recorded in this region during the Pliocene^37^,^38^, with a recent proposition that, during the mid-Pliocene, it may be mostly responding to changes in ventilation from the south, and so does not reflect changes in AMOC^39^.

In order to better assess early Pliocene changes in AMOC, we present benthic stable oxygen and carbon isotope data from ODP Site 1264 (2505 m depth, 29°S, 3°E), recently published by Bell *et al*.^33^, situated on the northern flank of Walvis Ridge in the Angola Basin of the Southeast Atlantic. Walvis Ridge forms an almost impassible barrier in most locations below ~3500 m and restricts water flow up to depths of ~2500 m, and, together with the Mid-Atlantic Ridge, acts to isolate the Angola Basin from deep-waters entering from the south^40^,^41^. As a result of this bathymetry, together with its position along a major export pathway of NADW^41^, below ~1500 m NADW dominates all depths in the Angola Basin. Furthermore, at ~18°S to the south of Walvis Ridge, Site 704 (also at ~2500 m depth) provides an important complementary “downstream” record for Site 1264, and allows an assessment of the relative strength of NADW presence in the Southeast Atlantic. Hence, Site 1264 is uniquely positioned to monitor fluctuations in NADW.

Using these new δ^18^O and δ^13^C data, together with published data from a number of other sites in the wider Atlantic (Fig. 3, Table 1), we assess the impact of the early Pliocene (4.7–4.2 Ma) shoaling phase of the CAS on AMOC.

Benthic foraminiferal δ^13^C records are a well-established tool for reconstructing circulation changes in the deep-ocean^42^,^43^, reflecting ambient nutrient concentration and the air-sea gas exchange history of source waters^44^. Meanwhile, δ^18^O records serve as a useful additional water-mass tracer^32^,^33^,^45^ as they document changes in the temperature and δ^18^O of ambient seawater, the latter of which co-varies with salinity changes. Hence, both δ^13^C and δ^18^O measurements are suitable tracers of chemical and physical changes in water-mass properties. An increase in NADW production is expected to increase δ^13^C values and/or reduce horizontal and vertical δ^13^C-gradients in the deep (>1 km depth) Atlantic^e.g.^^46^,^47^ as NADW has a higher δ^13^C signature than deep-waters originating from the south^48^. Concomitant changes in δ^18^O values are less straightforward to predict: it is possible that an increase in AMOC will be accompanied by an increase in δ^18^O, reflecting higher salinities and a low-latitude δ^18^O_seawater_ signature^33^, but this may be offset by the effects of warmer waters on δ^18^O during the equilibrium precipitation of calcite and/or local meltwater enriched in ^16^O from the north.

## Results

Timeseries of benthic δ^13^C and δ^18^O data from all Atlantic sites are presented in Figs 4 and 5, respectively, while site locations and data references are given in Table 1. The benthic δ^18^O stack of Lisiecki and Raymo^49^ (hereafter LR04) is also shown in Fig. 5 next to each record so that stratigraphic alignment can be seen. In order to directly assess the impact of the early Pliocene shoaling phase, we also compare average Atlantic deep-water conditions for time slices on either side of the period 4.7–4.2 Ma (Fig. 6).

Early Pliocene Atlantic δ^13^C records generally record high values, averaging between 0.5–1.0‰, directly comparable to values in the modern ocean (Fig. 4), and show little long-term trend. Interestingly, Site 1264 often records higher δ^13^C values than today, similar to modern core NADW values (~1‰). On the other hand, Sites 704 and 999 record low average values (~0–0.5‰) in early Pliocene, with Site 999 displaying a long-term increase from values averaging ~0.2‰ at the start of the Pliocene, rising to an average of ~0.65‰ by 3.7–3.6 Ma.

Atlantic δ^18^O records show a spread in average values similar in range to δ^13^C records. Sites 1264 and 704 have consistently higher δ^18^O values than the LR04 stack, while Sites 929 and 982 have consistently lower values. Long-term trends are generally small, with the notable exception of Site 925, which shows a large increase in δ^18^O at ~4.7 Ma.

Figure 6 shows average paired δ^18^O-δ^13^C anomalies for each site calculated by subtracting the mean ocean δ^18^O-δ^13^C changes, as recorded by ODP Site 849 in the deep Pacific^37^, for the intervals 5.0–4.7 Ma (Pre CAS shoaling) and 4.2–3.6 Ma (Post CAS shoaling). After mean ocean changes are removed, a significant increase (*p* = < 0.05 for student t-tests) in δ^13^C is only seen at Site 999 (Δδ^13^C = +0.18‰). δ^13^C changes in the remaining sites are small and not significant. Meanwhile, a significant and prominent shift in δ^18^O of +0.28‰ is observed for Site 925.

## Discussion

**Figure 2:**
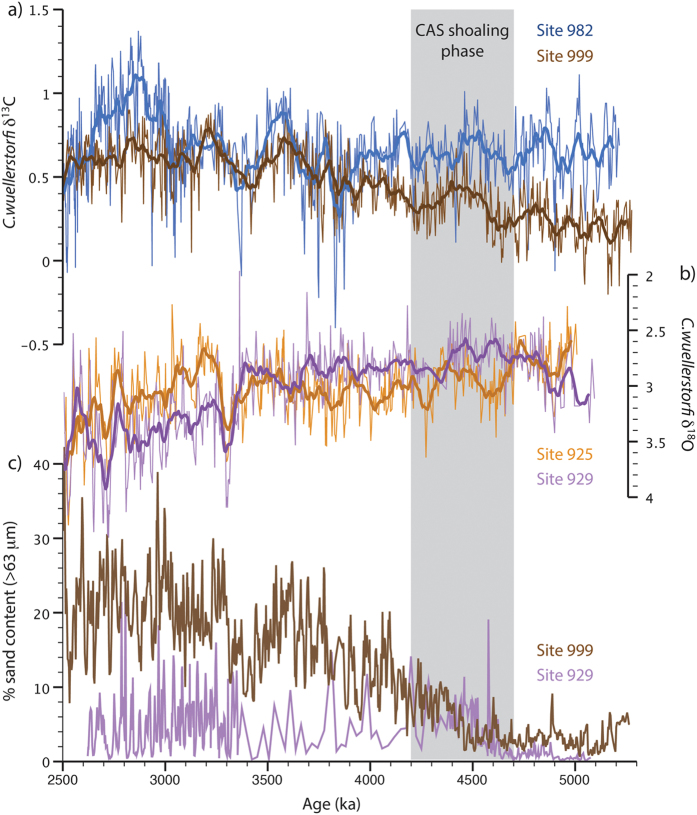
The main evidence for NADW changes in response to the early Pliocene shoaling of the CAS. (**a**) δ^13^C (‰ vs Vienna Pee Dee Belemnite) time series from Caribbean Site 999 and North Atlantic Site 982. The increasing δ^13^C values at Site 999 are interpreted to reflect a progressive increase in AMOC^3^; (**b**) δ^18^O timeseries (‰ vs Vienna Pee Dee Belemnite) from western equatorial Atlantic Sites 925 and 929. The reversal of δ^18^O gradients after ~4.7 Ma are interpreted to reflect an increase in temperature and salinity due to enhanced northward heat and salt transport^25^; (**c**) % sand content timeseries for Sites 999 and 929^3^. The increases in average and variability of % sand are interpreted to reflect enhanced carbonate preservation in response to an increase in dominance of low-corrosive NADW at both sites^3^. Bold lines represent 50 kyr running averages. References for isotopic data are given in Table 1.

The δ^13^C increase at Site 999, together with a concomitant increase in carbonate preservation^3^ (Fig. 2), is consistent with a southward shift in the boundary of Atlantic intermediate waters due to enhanced upper NADW formation in the region of the Labrador Sea, coeval with the progressive CAS shoaling phase. This interpretation is supported by modeling predictions^50^ and by the early Pliocene onset of drift deposits in sediments from the Labrador Sea (Site 646), indicating enhanced bottom water currents^51^. Rather than implying an overall restructuring of the deep and intermediate water circulation, however, the lack of substantial change elsewhere in the Atlantic Ocean suggests relatively local changes in water mass prevalence are being recorded in the Caribbean.

**Figure 3:**
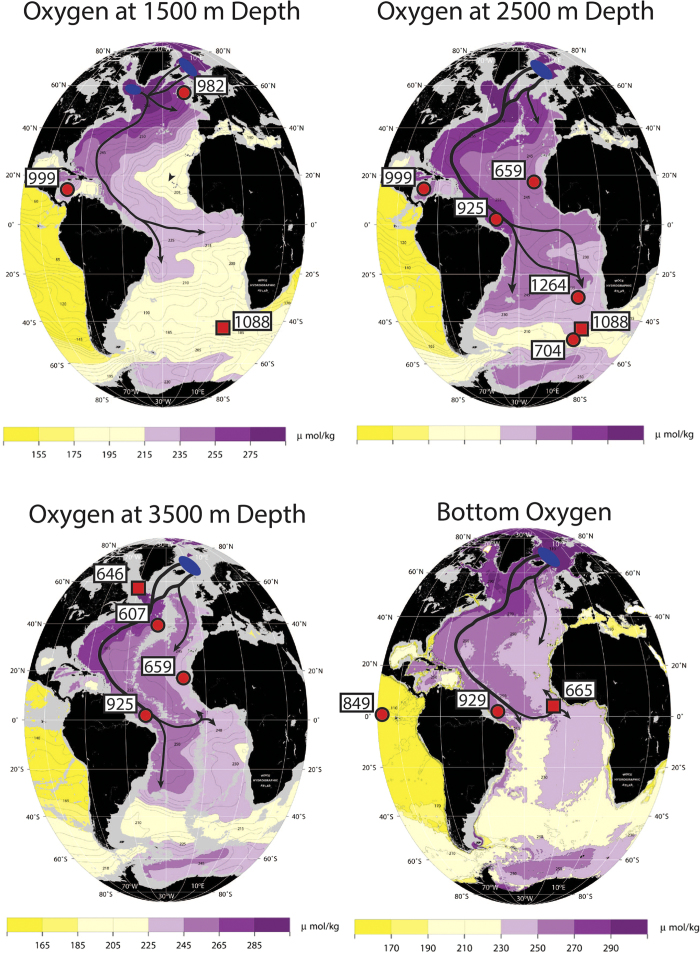
Dissolved oxygen concentration maps of the Atlantic at different depths (modified from the WOCE Atlantic Ocean Atlas 26 to show features of deep-water circulation and the locations of all sites referred to in the text), reflecting aspects of the chemical structure of the deep-Atlantic. Arrows depict general NADW circulation patterns and blue ovals indicate areas of NADW formation. Sites are displayed on maps according to their relevant depth (Table 1). Sites with isotopic data presented in Figs 4, 5, 6 plotted with a red circle, while other sites mentioned in the text are plotted with a red square. Site 999 is situated at 2828 m in the Caribbean, but is expected to record deep-waters entering across the Atlantic-Caribbean sills at ~1600–1900 m depth. High oxygen concentrations result from recent and prolonged contact with the atmosphere, while low oxygen concentrations result from microbial respiration of organic matter over time. NADW, with high oxygen concentrations, is a well “ventilated” water mass, in contrast with deep-waters entering the Atlantic from the south. These maps outline the modern dominance of NADW and highlight the importance of bathymetric constraints and deep-water pathways (e.g. the Deep Western Boundary Current versus interior pathways) in setting gradients in water-mass properties within the Atlantic.

Data from Site 1264 provide a reference for Site 704, forming a latitudinal transect (~28–47°S) of the Southeast Atlantic at ~2500 m depth. Since this region and depth is situated along a major export pathway for NADW^41^ and encompasses the modern boundary between NADW and Southern Source Water (SSW) (as reflected in Fig. 3), variations in Site 1264–704 δ^13^C-gradients gradients are expected to be sensitive to latitudinal movements in the extent of NADW, and hence reflect the strength of NADW formation. Throughout the interval studied, and with no apparent change in response to CAS shoaling, Site 1264–704 δ^13^C-gradients varied in the range of ~0.2–0.6‰, although sparse data from Site 704 indicate short periods of lower gradients. This is similar to the modern δ^13^C-gradient for this region, which is spatially variable and in the range of ~0.2–0.4‰^48^. δ^13^C–gradients between Site 1264 and sites in the North Atlantic are also similar to the modern situation, with the exception of Site 982–1264 gradients, which are even lower. Therefore, these observations provide a convincing basis for inferring that NADW formation during the early Pliocene was generally strong, with periods possibly stronger than today, and was seemingly unaffected by the CAS shoaling phase.

Previous studies have utilized δ^13^C data from Site 704 in order to reconstruct the relative strength of AMOC for intervals that encompass the early Pliocene^35^,^52^,^53^. Based on comparisons with available North Atlantic and Pacific records, and assuming a northern signal for δ^13^C changes, these studies speculated that NADW formation was already strong during the late Miocene, from ~6–7 Ma onwards, and that the early Pliocene progressive shoaling of the CAS (~4.7–4.2 Ma) had little impact. Similar conclusions were made with data from Site 1088^34^, which is at a slightly shallower depth in the same region (2080 m depth, 41°S, 14°E). Importantly, data from Site 1264^33^, which is uniquely located to record northern sourced deep-waters, substantiate these interpretations by demonstrating a positive N-S δ^13^C-gradient within the South Atlantic; a finding which is independent of remaining uncertainties in Southern Ocean ventilation ^e.g.^^39^.

Thus, given the confirmation of a northern δ^13^C-signal penetrating into the South Atlantic, we are able to quantitatively estimate the relative contribution of NADW versus SSW to waters bathing Site 1264. For a given site (*x*), this may be calculated using the equation of Oppo and Fairbanks^54^:


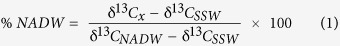


The δ^13^C_SSW_ is the end-member carbon isotopic value for SSW, δ^13^C_NADW_ is the end-member carbon isotopic value for NADW, and δ^13^C_x_ is the carbon isotopic value at site *x* in the Atlantic. For Site 1264, the calculation yields values of ~70%, both before and after the CAS shoaling phase (4.7–4.2 Ma), using NADW and SSW end-member values of Sites 607 and 704, respectively. By comparison, based on phosphate measurements^26^, this calculation indicates values of ~60–70% – depending on end-member values used – for the modern situation at Site 1264. This indicates that early Pliocene NADW formation was similar to the present, despite an open CAS. It should be noted, however, that this calculation relies on several assumptions, including the mixing of only two deep-water masses between all three sites, and that the end-member values of these water masses are properly captured by Sites 607 and 704.

The δ^18^O-gradient between Sites 925 and 607 (Fig. 6) records changes in mid-depth (3000–3500 m) waters between the western equatorial Atlantic and Northwest Atlantic, respectively (Fig. 3). Prior to the early Pliocene CAS shoaling phase, the large (~0.3‰) δ^18^O-gradient between these two sites points to differences in temperature and δ^18^O_seawater_ of the deep-waters flooding both locations, suggesting separate source regions in the North Atlantic (Fig. 6). Maintaining such a large δ^18^O-gradient at similar depths within the same basin would require a deep-water flow regime with little mixing between the core of the Deep Western Boundary Current (DWBC) (recorded by Site 925^25^) and the basin interior (recorded by Site 607^35^). Such a scenario may have existed due to weaker flow speeds, which promote more stable, laminar boundary flow^28^. Following from this, the absence of high-δ^13^C NADW with a low δ^18^O-signature being recorded elsewhere in the Atlantic could be explained if interior pathways were more important than the DWBC for deep-water export in the North Atlantic, as may be the case in the modern ocean^27^. Alternatively, it is also possible that the δ^18^O-shift at Site 925 is an artifact of measurement offsets between laboratories, which can be as large as 0.3‰^55^. However, the reversal of the Site 929–925 δ^18^O-gradient should remain a robust result, as both sets of data were measured at the same laboratory^25^.

**Figure 4:**
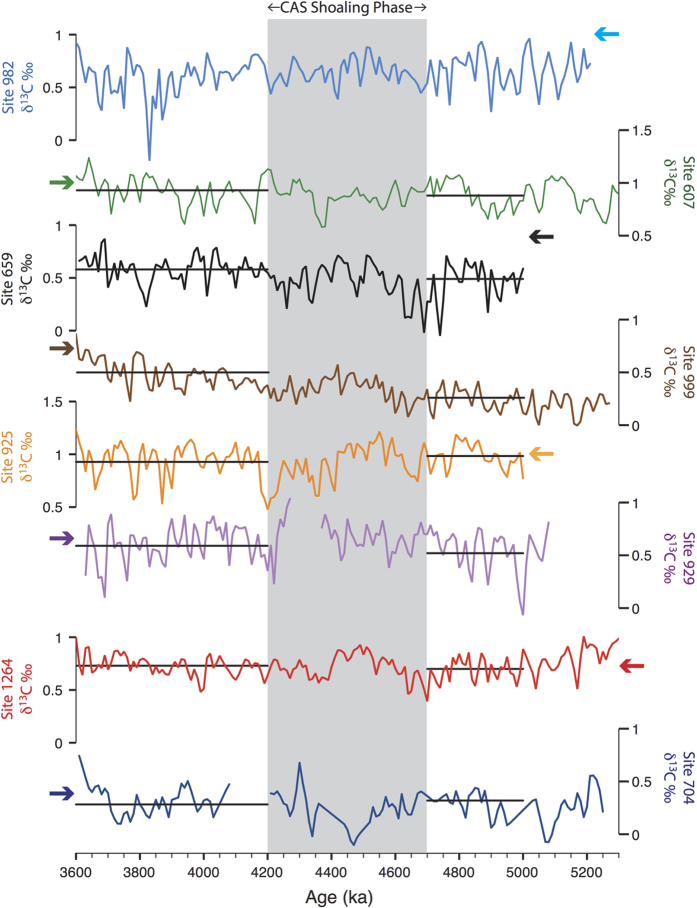
Time series of δ^13^C data (‰ vs Vienna Pee Dee Belemnite) from all sites shown in Fig. 1. Data has been resampled at common 10 kyr time steps (linear integration). Horizontal black lines show average δ^13^C values for the time slice intervals 5.0–4.7 Ma (Pre CAS shoaling) and 4.2–3.6 Ma (Post CAS shoaling). Arrows indicate approximate modern δ^13^C values for each site location^48^. Note that data for Site 607 has been resampled at 10 kyr intervals, as is available from^32^, while data from Site 704 prior to 4.2 Ma is displayed as symbols in order to highlight the low resolution and stratigraphic uncertainty. Data references are given in Table 1.

**Figure 5:**
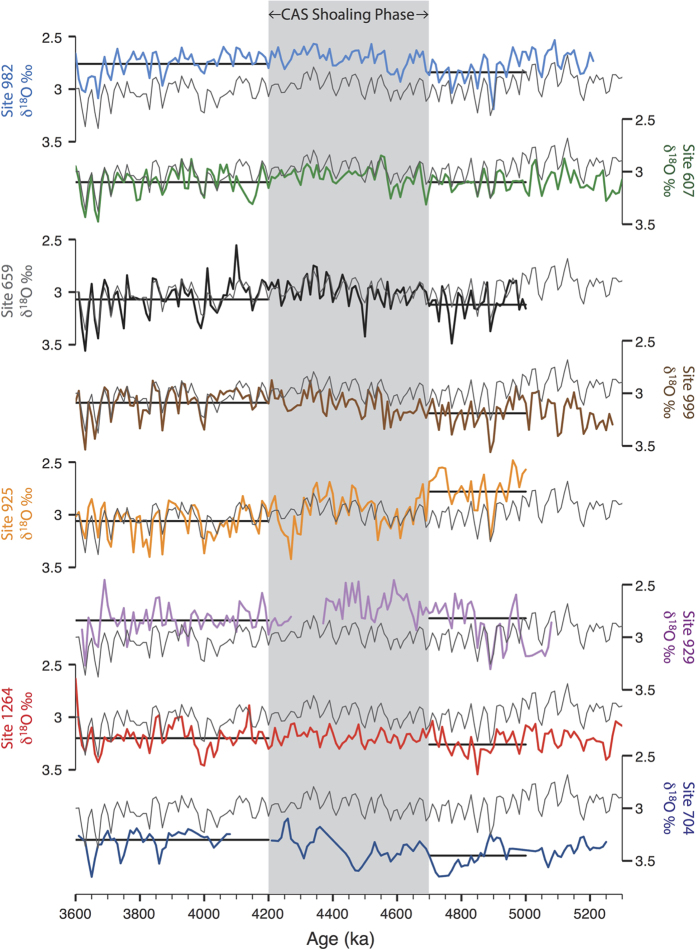
Time series δ^18^O data (‰ vs Vienna Pee Dee Belemnite) from all sites shown in Fig. 1 (thick colored lines). Data has been resampled at common 10 kyr time steps (linear integration). Horizontal thick black lines show average δ^18^O values for the time slice intervals 5.0–4.7 Ma (Pre CAS shoaling) and 4.2–3.6 Ma (Post CAS shoaling). The LR04 stack^38^ is shown along side each record (thin gray lines), on the same scale, to show stratigraphic alignment (see Methods). Note that data from Site 1264 has been resampled at 3 kyr intervals so that stratigraphic alignment can be clearly seen, while Site 704 prior to 4.2 Ma is displayed as symbols in order to highlight the low resolution and stratigraphic uncertainty. References for isotopic data are given in Table 1.

**Figure 6:**
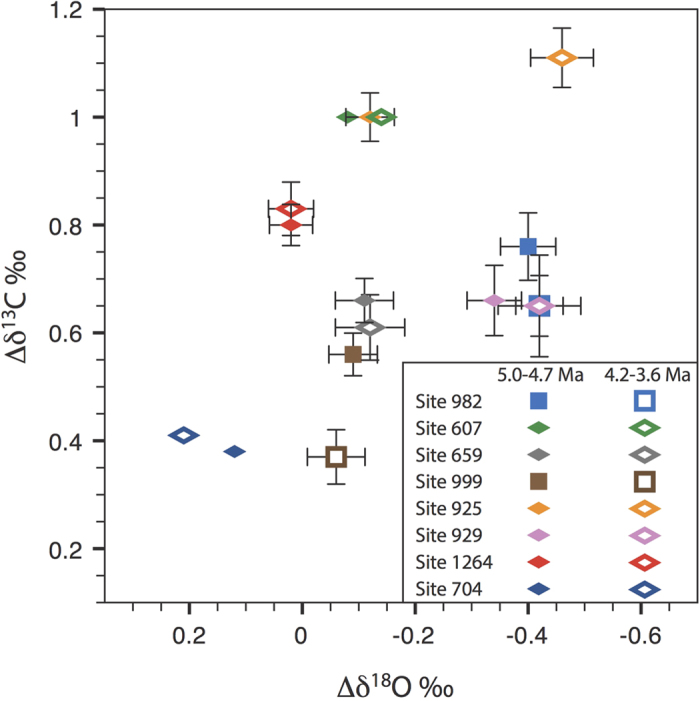
Average δ^13^C and δ^18^O values (both plotted in ‰ vs Vienna Pee Dee Belemnite) for the time slice intervals 5.0–4.7 Ma (Pre CAS shoaling) and 4.2–3.6 Ma (Post CAS shoaling) plotted as anomalies with respect to changes at Pacific Site 849 (see text). Square symbols indicate sites recording ocean water masses at intermediate depths (1000–2000 m) while diamond symbols indicate sites recording water masses in the deep ocean (>2000 m). Error bars are 2σ combined error on the difference, Due to poor time resolution for Site 704 and lack of original time series data for Site 607, no error bars are shown for these sites. Note that errors serve only as an indication of the confidence in mean values calculated. Data references are given in Table 1.

Billups *et al*.^25^ recognized the early Pliocene δ^18^O increase at Site 925 and the resulting reversed δ^18^O-gradient with underlying waters at Site 929 (Fig. 2). Based on the requirement of a stable vertical density structure, they concluded that Site 925 deep-waters must have become warmer and saltier between ~4.2–3.7 Ma. Figures 4 and 5 indicate that deep-water conditions at Site 925 were unusual prior to 4.7 Ma and that, subsequently, the prevalence of deep-waters sourcing other mid-depth (2500–3500 m) sites (i.e. Sites 607, 659 & 1264) increased. The heavier δ^18^O-signature of deep-waters recorded at Site 925 may also reflect a strengthened low-latitude surface-water signal^33^, in line with inferences of an increase in temperature and salinity.

**Figure 7:**
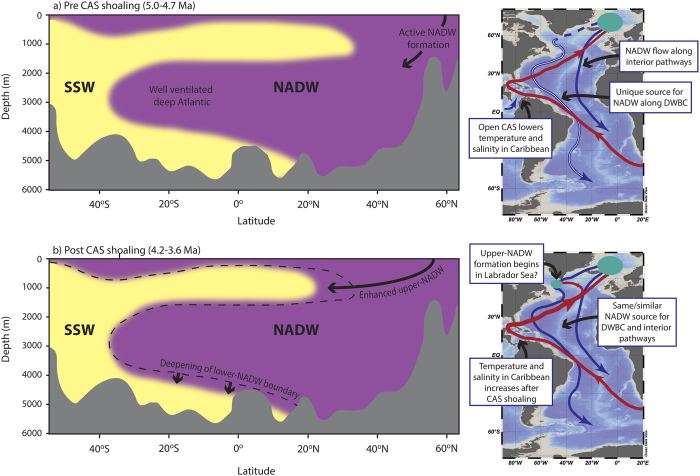
A schematic of Atlantic deep-water changes in response to the early Pliocene CAS shoaling phase (~4.7–4.2 Ma). The dashed line in b) shows the boundary between Southern Sourced Water (SSW) and North Atlantic Deep Water (NADW) prior to CAS shoaling, for reference. The red arrows on the map sections indicate the northward surface flow of warm water, which was possibly slightly enhanced after CAS shoaling. Green circles indicate areas of deep-water formation in the North Atlantic. Maps generated using Adobe Illustrator.

While stable isotope evidence suggests that deep-water change in response to the progressive CAS shoaling phase (4.7–4.2 Ma) was restricted to a strengthening of upper NADW formation and an altered flow regime of the DWBC, sedimentary evidence of increased carbonate preservation suggests a further increase in the prevalence of low-corrosive NADW. An increase in the sedimentary carbonate fraction from ~0% to ~40% is seen during the early Pliocene in the eastern North Atlantic (Site 665^56^, 4755 m). Given the highly nonlinear relationship between %CaCO_3_ and dissolution, however, this may only reflect small changes in preservation. Meanwhile, in the western equatorial Atlantic (Site 929, 4350 m), an abrupt increase in % sand fraction variability is seen after ~4.6 Ma^3^ (Fig. 2), likely relating to phases of enhanced carbonate preservation^24^. While this is not reflected in average values (Fig. 7), it is consistent with an increase in peak δ^13^C values at Site 929^25^,^57^. These observations may be explained by a deepening of the lower boundary of NADW, such that preservation conditions at Site 929 became sensitive to variations in AMOC after CAS shoaling. In conjunction with evidence for enhanced deep-water formation in the Labrador Sea, this is remarkably consistent with modeling predictions by Mikolajewicz & Crowley^50^.

## Conclusions

Figure 7 provides an illustrative summary of deep-water changes inferred from the stable isotope and carbonate preservation evidence described above. Prior to the early Pliocene CAS shoaling phase (Fig. 7a), we find strong evidence for a well-ventilated deep Atlantic as far a ~28^o^S, suggesting that NADW formation was active despite the open gateway. Low δ^13^C-gradients between various sites across the Atlantic indicate that deep-water circulation was rigorous, comparable to and frequently stronger than today. In particular, our new data confirm the dominance of NADW in the Southeast Atlantic. However, upper-NADW formation in the Labrador Sea was reduced and, taking changes in δ^18^O data at face value, deep-waters within the DWBC were distinct from those filling the wider Atlantic, implying limited mixing with deep-waters in the basin interior, possibly due to low flow speeds.

The early Pliocene progressive CAS shoaling phase had a limited effect on the overall geometry of Atlantic deep-waters (Fig. 7b). This is indicated by the negligible changes to the isotopic signature of deep-waters at most Atlantic sites. Instead, changes were restricted to a few key regions: Upper-NADW formation in the Labrador Sea began a long-term increase^3^, while deep-waters sourcing the DWBC became more similar to those filling the basin interior, possibly reflecting an increase in temperature and salinity^25^ in response to enhancements in the Gulf Stream. Meanwhile, the depth of the lower boundary of NADW deepened slightly, increasing carbonate preservation in the deep North Atlantic (>4000 m).

We conclude that the lack of change in large parts of the Atlantic Ocean demonstrates that the early Pliocene shoaling phase of the CAS had little impact on AMOC and, therefore, climate. Our data corroborate data and modeling studies that propose significant NADW flow existed with an open CAS^e.g.^^34^,^35^,^46^,^50^,^52^,^58^ and suggest that large scale changes in Atlantic deep-water circulation, if any, would have occurred in response to earlier and deeper shoaling phases of the CAS, possibly during the late Miocene^19^.

## Methods

10 cm^3^ samples were taken from Site 1264 cores at 1.5 cm intervals, using the shipboard splice to create a meters composite depth (mcd) scale. For the large majority of Site 1264 samples (30.01–57.45 mcd = 3.715–5.300 Ma), processing and measurements were carried out at the University of Florida. Benthic species, *Cibicidoides wuellerstorfi*, were picked from the >150 μm size fraction. The foraminifera were then cleaned in an ultrasonic bath to remove fine-grained particles and soaked in 15% H_2_O_2_ to remove surface organic contaminants prior to analysis. The number of specimens of *C. wuellerstorfi* varied from 1 to 4 and foraminiferal tests were crushed before analysis. The foraminiferal calcite was reacted in 70 ^o^C orthophosphoric acid using a Kiel III carbonate preparation device. Evolved CO_2_ gas was measured online with a Finnigan-MAT 252 mass spectrometer.

For samples between 27.80–30.01 mcd (=3.600–3.715 Ma), samples were processed and measured at Vrije Universiteit, Amsterdam. Measurements were performed on 1–3 *C. wuellerstorfi*, selected from the >200 μm size fraction, using a Finnigan 251 Gas Source mass spectrometer equipped with Kiel type automated carbonate extraction line.

All stable isotope results are reported relative to Vienna Pee Dee Belemnite and calibrated using in house standards that are correlated to the international standard, NBS19. Analytical precision for both δ^18^O and δ^13^C is better than ±0.1‰. For all sites assessed, δ^18^O values are adjusted by +0.64‰ to account for disequilibrium with surrounding seawater^59^.

Systematic inter-laboratory offsets are a possible artifact in δ^18^O records, generated by pooling data from different labs. Indeed, our experience from a small number of duplicate measurements taken between different laboratories indicates potential offsets of up to 0.1–0.2‰, in line with previous findings of up to 0.3‰^55^. However, given practical limitations on the number of duplicate measurements possible, including limited availability of *C. wuellerstorfi* specimens, such offsets are difficult to correct for. Hence, we are aware of potential offsets of <0.3‰.

All age models used in this study were derived from the alignment of δ^18^O time series to the global benthic δ^18^O stack of Lisiecki and Raymo^49^ (the LR04 stack), as described in Bell *et al*.^33^ and shown in Fig. 5. The low resolution and short hiatuses present in early Pliocene data from Site 704, however, prevented reliable alignment, so the original age model^36^ was used. Averages calculated from these data may thus be affected by age model uncertainties and missing information.

## Additional Information

**How to cite this article**: Bell, D. B. *et al*. Atlantic Deep-water Response to the Early Pliocene Shoaling of the Central American Seaway. *Sci. Rep*. **5**, 12252; doi: 10.1038/srep12252 (2015).

## Figures and Tables

**Figure 1:**
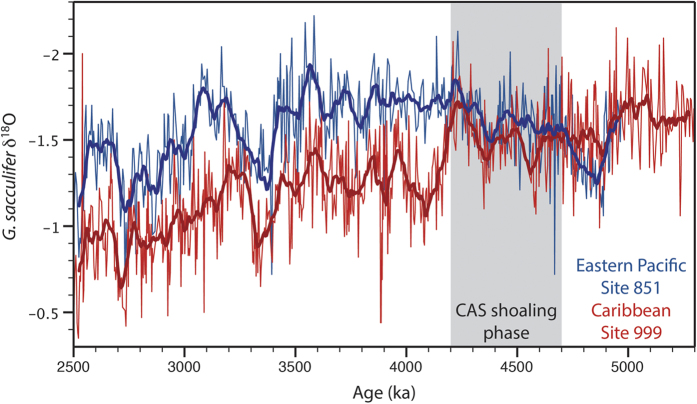
Surface water (planktic) δ^18^O records (‰ vs Vienna Pee Dee Belemnite) from the Caribbean (Site 999)^21^ and Pacific (Site 849)^62^ showing the emergence of a permanent gradient between ~4.7–4.2 Ma, interpreted to reflect a major shoaling phase of the CAS^3^. Bold lines represent 50 kyr running averages.

**Table 1:** All site locations, water depth, average age resolution for the period 5.3–3.6 Ma, and sources of isotopic data used in this study.

Site	Location	Water Depth (m)	Av. Age Resolution (ka)	References
982	58^o^N, 16^o^W	1145	4.6	35
646	58°N, 48°W	3450	*No isotopic data presented*	
607	41°N, 33°W	3430	5.0	35
659	18°N, 21°W	3070	5.6	60
665	3°N, 20°W	4755	*No isotopic data presented*	
999	13°N, 79°W	2828	3.9	3
929	6°N, 44°W	4370	5.9	57,61
925	4°N, 43°W	3040	4.19	57,61
1264	29°S, 3°E	2505	1.5	33
1088	42°S, 15°E	2080	*No isotopic data presented*	
704	47°S, 7°E	2530	11.5	36
849	0°N, 110°W	3840	5.2	37
